# Trunk- and branch- data derived radial increments of *Pinus brutia* Ten., *Cupressus sempervirens* L., and *Quercus pubescens* Willd. subsp. *pubescens* from Crete for dendro- and anthraco-typological analysis

**DOI:** 10.1016/j.dib.2025.112307

**Published:** 2025-11-23

**Authors:** Léane Levillain, Mélanie Saulnier, Alexa Dufraisse, Laurent Larrieu, Maria Ntinou, Vanessa Py-Saragaglia

**Affiliations:** a GEODE UMR 5602 CNRS-Université de Toulouse, Maison de la recherche, 5 allées Antonio Machado, Toulouse Cedex 9 31058, France; b IRAMAT-LMC UMR 7065 CNRS-UTBM Belfort-Sévenans, Université de Technologie de Belfort-Montbéliard, Campus de Sévenans 90010 Belfort cedex, France; c BIOARCH UMR 7209 CNRS-MNHN, Muséum national d’Histoire naturelle, CP 56 - 57 rue Cuvier 75005 Paris, France; d DYNAFOR UMR 1201 INRAE, Université de Toulouse, 24 Chemin de Borde-Rouge Auzeville CS 52627 31326 Castanet Tolosan Cedex, France; e CNPF-CRPF Occitanie, France; f School of History and Archaeology, Aristotle University of Thessaloniki, Faculty of Philosophy 54124 Thessaloniki, Greece

**Keywords:** Tree-ring-width, Branch, Trunk, Crete, Timber, Turkish pine, Mediterranean cypress, Downy oak

## Abstract

This dataset provides tree-ring width series for three key Mediterranean tree species that were commonly used in Aegean Bronze Age architecture: *Pinus brutia* Ten. (Turkish pine), *Cupressus sempervirens* L. (Mediterranean cypress), and *Quercus pubescens* Willd. subsp. *pubescens* (Downy oak). The dataset, which was collected from well-characterised forest stands in eastern Crete, includes measurements from both trunk and branch slices. A subset of branch samples was experimentally carbonised to quantify radial shrinkage, a critical correction factor for analysing archaeological charcoal. Together, these data offer the first species-specific anatomical references for distinguishing trunk- and branch-derived wood on the basis of tree-ring width. The dataset supports applications in dendrochronology, anthracology, and wood-use reconstruction, including the identification of selective logging or fuel-management practices. It also includes R scripts for applying a generalised linear model to identify the anatomical origin of archaeological wood remains. Chronologies span up to 155 years for cypress, 130 years for pine, and 55 years for oak. While the pine and oak trunk chronologies have provided reliable crossdating, the cypress chronologies and branch datasets are more suitable for anthraco-typological analysis, i.e. for differentiating branch and trunk wood based on anatomical and growth-ring features. Nevertheless, correcting the cypress series on the basis of a robust chronology will provide an insightful growth ring chronology for Crete.

Specifications Table

**Table:** 

Subject	Earth & Environmental Sciences
Specific subject area	Dendrotypological references (branches and trunks on Turkish pine, Mediterranean cypress and Downy oak from living trees in Crete) used for antracological analysis.
Type of data	Tables (.csv and .xlsx format) Heidelberg Format Ring Width files (.fh or .rwl format) R.files (.R) 4 figures
Data collection	This dataset provides ring-width series for three key Mediterranean tree species used in Aegean Bronze Age architecture: *Pinus brutia, Cupressus sempervirens*, and *Quercus pubescens*. Samples were collected from well-characterised forest stands in eastern Crete and include both trunk cores and branch slices. A subset of branch samples was experimentally carbonised to quantify radial shrinkage, a critical parameter for correcting archaeological charcoal measurements.
Data source location	The data were collected in Crete (in the Kroustas, Kritsa and Kera forests) They are now stored in the GEODE laboratory (CNRS UMR 5602, Toulouse, France).
Data accessibility	Repository name: https://data.indores.fr Data identification number: 10.48579/PRO/W314TB Direct URL to data: https://doi.org/10.48579/PRO/W314TB
Related research article	NA

## 1 Value of the Data

• The datasets provide new reference chronologies of tree-ring growth for Turkish pine (*Pinus brutia*) and Downy oak (*Quercus pubescens* Willd. subsp. *pubescens*) in Crete.
• The chronology for Mediterranean cypress (*Cupressus sempervirens*) requires further crossdating with robust regional chronologies to confirm its reliability and establish it as a new reference for the area.
• The datasets include the first wood anatomical references for distinguishing trunk and branch wood in these three key taxa, based on growth-ring patterns, thereby providing a practical toolbox for dendro- and anthraco-typological applications in archaeological contexts.
• Based on branch wood, ring-width measurements taken before and after experimental carbonisation, enable us to assess the radial shrinkage due to charring.
• The dataset further provides valuable reference data for climate studies in the eastern Mediterranean, as the tree-ring chronologies cover the last 130 years for Turkish pine, 155 years for Mediterranean cypress, and 55 years for downy oak.

## 2 Background

Timber was a crucial element of Aegean Bronze Age architecture, in both Minoan and Mycenaean traditions. Excavations and iconographic evidence show its use in walls, roofs, and vertical structural reinforcements [[1], [2], [3], [4], [5]]. Since the 1980s, anthracological studies have revealed a wide range of taxa, with preferences for native conifers (pines, cypress) and for olive and oaks among broadleaves [[6], [7], [8], [9], [10], [11], [12]]. Yet significant gaps persist regarding: (i) the proportion of branch versus stem wood, (ii) the size and growth form of selected material, and (iii) the silvicultural practices shaping exploited landscapes.

Within the TiMMA project, we aimed to: (i) develop a method for identifying the position of wood within the tree (trunk vs branch) using growth-ring patterns, and (ii) assess the effects of carbonisation on radial shrinkage (see supplementary file 1a). To build such references, we focused on Crete, where anthracological data are most abundant (see supplementary file 1b), and selected three key species: Turkish pine (*Pinus brutia* Ten., 1811), Mediterranean cypress (*Cupressus sempervirens* L., 1753), and Downy oak (*Quercus pubescens* Willd. subsp. *pubescens,* 1796), also referred to as *Quercus brachyphylla* Kotschy, 1859 [[13], [14]].

## 3 Data Description

The directory associated with this article consists of four folders (some with subfolders) and one text file, and is available at: https://doi.org/10.48579/PRO/W314TB

• **(1) Individual Series Folder:** This folder contains the tree-ring width series used for constructing chronologies (.fh files). It is organised into two levels of subfolders.
  • The first level of subfolders indicates the measured taxon (Turkish pine, Mediterranean cypress, or Downy oak).
  • The second level of subfolders within each taxon indicates whether the measurement was taken from a branch or a trunk.
• **(2) Tables Folder (.xlsx; .csv):** This folder contains several tables with 'Read_me' sheets that explain the variables and abbreviations in each table. Seven tables are shown in this paper, and the “Metadata” table and “Table 8” are available here: https://data.indores.fr:443/api/access/datafile/30827; https://data.indores.fr:443/api/access/datafile/29440
  • **Metadata** explain all that the dataset contains and its structure.
  • Table 1 presents the geographical characteristics of the analysed sites, including the forest name, the plot number, the vegetation zone, the context and substratum, the forest stand and the herbaceous flora.

**Table 1:** Geographical characteristics of the analysed sites, including the forest name, the plot number, the vegetation zone, the context and substratum, the forest stand and the herbaceous flora.

Site	N°Plot	Stage	Context & Substratum	Forest stand	Non-tree taxa
Kritsa	1	Upper mesomediterranean /supramediterranean (950 m); Acero-cupression	Hard carbonate rock, conglomerate, alveolar erosion facies; dry valley border	Open grazed forest of *Cupressus sempervirens* (Cs), *Quercus coccifera* (Qc) & *Acer sempervirens* (As); numerous coppiced forms of Qc; some Qc with dbh >100 cm; several Cs are multi-stemmed; no stumps	*Cyclamen cretica*, short pink orchid with a large spur, spiny Euphorbia
Kritsa	2	Upper mesomediterranean /supramediterranean (890 m); Acero-cupression	Hard carbonate rock, conglomerate, alveolar erosion facies; dry valley border	Open grazed forest of *Cupressus sempervirens* (Cs), *Quercus coccifera* (Qc) & *Acer sempervirens* (As); numerous coppiced forms of Qc; some Qc with dbh >100 cm; several Cs multi-stemmed; some branches cut with axes; large pollarded Qc; Cs also grazed, some bonsai-like; no stumps	*Cyclamen cretica*, short pink orchid with a large spur, spiny *Euphorbia, Paeonia* sp.
Kritsa	3	Upper mesomediterranean /supramediterranean (870 m); Acero-cupression	Hard carbonate rock, conglomerate, alveolar erosion facies; very thin soil (but cracked?); % of surface rock >75 %	Open grazed forest of *Cupressus sempervirens* (Cs) and *Quercus coccifera* (Qc); two-layered stand: (i) Tree layer with multiple cohorts (Cs: 10–25 and 45–50; Qc: 10–25, 40–50, and >100); (ii) Sub-shrub layer with pruned forms; Qc all pollarded, some Cs multi-stemmed; no stumps	
Kroustas	1	Upper mesomediterranean (780 m); Irido cretensis-Pinetum brutiae		Pre-woodland of *Pinus brutia* (Pb) (<10m?) & *Acer sempervirens* (grazed form) and *Quercus coccifera* (grazed form); all pines have axe notches ("ted" type); several coppicing stages; very few standing deadwood and no deadwood on the ground; large lower branches of pines cut; shrub undergrowth heavily browsed (0–2 m, ∼80 % cover); many enclosures and free-ranging goats	*Dracunculus vulgaris, Sarcopoterium spinosus, Calicotome* sp*., Asphodeline lutea, Rhamnus lycioides*
Kroustas	2	Upper mesomediterranean /supramediterranean (820 m); Irido cretensis-Pinetum brutiae		*Pinus brutia* regrowth on an old complex of small agricultural terraces (100–500m²) with a threshing area; mean tree height ∼15 m; Pb associated with *Quercus coccifera* (both tree and grazed forms); all oaks pollarded at ∼2 m; Pb dbh 30–50 cm with some larger (∼80 cm); some Pb with axe notches ("ted" type)	*Cheilanthes acrostica, Ceterach officinale*
Kroustas	3	Upper mesomediterranean /supramediterranean (824 m); Irido cretensis-Pinetum brutiae	Entirely carbonate conglomerate	Dense stand (∼70 %) of *Pinus bruti*a with axe notches ("ted" type), associated with pollarded *Quercus coccifera*; no terraces within 50 m, but situated in a terraced landscape; many lower branches cut with axes on Pb and Qc	*Pinus brutia*, tree and grazed forms of *Quercus coccifera, Acer sempervirens* (grazed form), *Cyclamen creticum* ("colchicum"), *Cistus creticus, Cistus salvifolius, Erica manipuliflora, Erica arborea, Osyris alba, Hypericum empetrifolium, Anthoxanthum odoratum, Pistacia terebinthus, Lonicera etrusca, Calicotome* sp*., Olea europaea*
Kera	1	mesomediterranean (540 m); Cyclamino-Quercion illicis	Pelitic schists; some Permo-Triassic pelitic layers; presence of a humus form	Sparse coppice of *Quercus coccifera* and *Q. pubescens*, low dominant height, on old terraces (former fruit orchards?)	**Flora**: *Geranium robertianum, Galium aparine, Erica manipuliflora, Crataegus monogyna (azarella), Cistus creticus, Tamus communis creticus, Cyclamen* sp*., Cistus salviifolius, Acanthus spinosus, Dittrichia viscosa, Sarcopoterium spinosum, Gallium aparine, Rubia peregrina, Oxalis pes-caprae*
Kera	2	mesomediterranean (540 m); Cyclamino-Quercion illicis	Cultivated terraces & olive trees; Quercus pubescens on the edges; fairly thick colluvial soil	Sparse coppice of *Quercus coccifera* and *Q. pubescens*, low dominant height, on old terraces (former fruit orchards?)	**Flora**: *Geranium robertianum, Galium aparine, Erica manipuliflora, Crataegus monogyna (azarella), Cistus creticus, Tamus communis creticus, Cyclamen* sp*., Cistus salviifolius, Acanthus spinosus, Dittrichia viscosa, Sarcopoterium spinosum, Gallium aparine, Rubia peregrina, Oxalis pes-caprae*
Kera	3	mesomediterranean (560 m); Cyclamino-Quercion illicis	Terrace borders currently cultivated in olives; acidic pelitic schist; thick soil but rich in coarse elements, humus on the surface	Sparse coppice of *Quercus coccifera* and *Q. pubescens,* low dominant height, on old terraces (former fruit orchards?)	*Cistus creticus, Cistus salvifolius, Erica manipuliflora, Osyris alba, Hypericum empetrifolium, Asparagus* sp.
  • Table 2 presents the characteristics of the sampled trees and plot-level attributes: plot ID, tree ID, geographic coordinates (latitude, longitude; WGS84), altitude (m a.s.l), diameter at breast height (DBH) (cm), total tree height (m), competition index (count of neighbouring trees whose upper branches overlap or mingle with those of the target tree), as well as the number of samples collected (core; branch), management form, and use marks.

**Table 2:** Characteristics of tree sampling.

Site/plot	Name of tree	Coordonates		asl. (m)	dbh (cm)	htot (m)	Competition Index (No)	Others	No core 2024	No core 2023	No branch 2024	No branch 2023
		Lat.	Long.									
Kritsa_1	Cs120	35.156533978879452	25.593630038201809	1021	38	9	0	single stem	1	1	1	5
Kritsa_1	Cs83	35.156774958595634	25.593809997662902	1016	26	10	0	single stem	1	1		2
Kritsa_1	Cs51	35.156995989382267	25.593865988776088	935	51	12	2	single stem	1	1		5
Kritsa_1	Cs82	35.156834973022342	25.593879986554384	937	26	8	0	single stem	1	1		3
Kritsa_1	Cs42	35.157036976888776	25.593675971031189	941	42	13	1	several stems	2		3	
Kritsa_2	Cs37	35.157637959346175	25.594394970685244	911	37	10	1	single stem	2		3	
Kritsa_2	Cs13	35.157777015119791	25.594401005655527	909	13	10	2	single stem	2		3	
Kritsa_2	Cs26	35.157856978476048	25.594416009262204	908	26	10	1	single stem	2		3	
Kritsa_2	Cs35	35.157812973484397	25.594647014513612	908	35	11	0	single stem	2		3	
Kritsa_2	Cs25	35.157896960154176	25.594871984794736	909	25	7	3	single stem	3		3	
Kritsa_3	Cs52	35.158389983698726	25.594891011714935	895	52	12	1	single stem	2		3	
Kritsa_3	Cs15	35.15842099674046	25.594909032806754	898	15	8	0	single stem	3		3	
Kritsa_3	Cs21	35.158498026430607	25.59498799033463	995	21	9	0	single stem	2		3	
Kritsa_3	CS43	35.158457038924098	25.595065020024776	995	43	12	2	single stem but small branches cut by axe	2		3	
Kritsa_3	Cs26	35.15850598923862	25.595169039443135	899	26	7	2	several stems	2		3	
Kroustas_0	Pb208	35.122922966256738	25.632279999554157	840	66	Na	Na			1		4
Kroustas_0	Pb156	35.122922966256738	25.632279999554157	840	50	Na	Na			1		3
Kroustas_0	Pb279	35.124400025233626	25.625932971015573	893	89	Na	Na			1		4
Kroustas_1	Pb330	35.11886402964592	25.635982034727931	804	105	12	0	"tèdes"	1		3	
Kroustas_1	Pb303	35.119097968563437	25.636457037180662	811	96	15	0	"tèdes"	1	2		3
Kroustas_1	Pb162	35.11899302713573	25.636313958093524	811	52	8	0	"tèdes"	2		4	
Kroustas_1	Pb180			816	57	13	0	"tèdes" + mistletoe	2		2	
Kroustas_1	Pb170	35.11931799352169	25.636503975838423	822	54	8	1	"tèdes" + mistletoe; twin	2		3	
Kroustas_2	Pb136	35.12159401550889	25.634096022695303	836	43	15	2	no "tède", no mistletoe; twin	2	1	3	
Kroustas_2	Pb46			841	15	7	2	no "tède"; 1 subcortical yellow scorpion; snag decay stade 2	1		3	
Kroustas_2	Pb124	35.121589992195368	25.634039025753736	835	39	12	1	no "tède", no mistletoe	2		3	
Kroustas_2	Pb218	35.121910013258457	25.63325397670269	827	69	14	1		1	1		3
Kroustas_2	Pb132	35.121494019404054	25.63428302295506	835	42	13	1	twin	1	1		3
Kroustas_3	Pb133	35.123291015625	25.631786976009607	844	42	6	3	"tèdes"; no mistletoe	2		3	
Kroustas_3	Pb131	35.123282968997955	25.631960984319448	844	42	13	3	no "tède", twin	2		3	
Kroustas_3	Pb94	35.123443985357881	25.631952015683055	839	30	8	2	no "tède", no mistletoe	2		3	
Kroustas_3	Pb147	35.123675996437669	25.632025022059679	815	47	16	1	no "tède", mistletoe	2		3	
Kroustas_3	Pb148	35.123239969834685	25.631842967122793	844	47	7	1	"tèdes" + mistletoe	2		3	
Kera_1	Qp19dbh	35.229813018813729	25.459387991577387	620	19	5	2	coppice with 2 sprouts	2		3	
Kera_1	Qp20dbh	35.229802038520575	25.459450017660856	620	20	4	2	coppice with 4 sprouts	2		3	
Kera_1	Qp39dbh	35.229780999943614	25.459569040685892	619	39	6	2	coppice with 2 sprouts	2		3	
Kera_1	Qp18dbh	35.229714028537273	25.459598964080215	623	18	6	2	coppice with 2 sprouts	2		3	
Kera_1	Qp35dbh	35.22987999022007	25.459723016247153	613	35	6	0	coppice with 1 sprout	2		3	
Kera_2	Qp37dbh	35.228626979514956	25.461553959175944	608	37	8	0	old coppice	2		3	
Kera_2	Qp23dbh	35.228503011167049	25.461591007187963	616	23	5	3	coppice with 2 sprouts	2		3	
Kera_2	Qp14dbh	35.228530000895262	25.461788987740874	615	14	7	2	coppice with 1 sprout	2		3	
Kera_2	Qp41dbh	35.228447020053864	25.461842967197299	625	41	8	2	coppice with 3 sprouts	2		3	
Kera_2	Qp27dbh	35.228521032258868	25.461794016882777	622	27	9	4	coppice with 4 sprouts	2		3	
Kera_3	Qp53dbh	35.227684015408158	25.46137098222971	629	53	7	0	free-standing with low cut branches	/		3	
Kera_3	Qp16dbh	35.2276870328933	25.461144000291824	629	16	5	3	coppice with 3 sprouts	2		3	
Kera_3	Qp09dbh	35.227650990709662	25.461079962551594	625	9	4	3	free-standing	2		3	
Kera_3	Qp22dbh	35.227746963500977	25.461024977266788	626	22	5	2	free-standing + pollarding	2		3	
Kera_3	Qp24dbh	35.22809699177742	25.460849041119218	624	24	8	2	free-standing	2		3	

“Tèdes”: Pyrenean term for the traces of sapwood strips removed from the base of trunks; these resin-impregnated wood shavings were once used for lighting huts and lighting fires.
  • Table 3 shows the shrinkage calculation for each species, as well as the diameter before and after carbonisation.

**Table 3:** Measure of diameter before and after carbonisation.

	Diameter (cm) before carbonisation					Diameter (cm) after carbonisation					Percentage of shrinkage				
Kritsa	Branch 1	Branch 2	Branch 3	Branch 4	Branch 5	Branch 1	Branch 2	Branch 3	Branch 4	Branch 5	Branch 1	Branch 2	Branch 3	Branch 4	Branch 5
Cs_82	3	4,3	1,4	/	/	1,9	3,5	1	/	/	37 %	19 %	29 %	/	/
Cs_160	4,1	8,2	2,3	3,3	5,6	3,2	6	1,7	2,4	4,5	22 %	27 %	26 %	27 %	20 %
Cs_120	3	3,8	4,4	1,4	2,3	2,3	2,8	3,5	1,2	2	23 %	26 %	20 %	14 %	13 %
Cs_83	4,5	5,6	/	/	/	3,5	5	/	/	/	22 %	11 %	/	/	/

Kroustas	Branch 1	Branch 2	Branch 3	Branch 4	Branch 5	Branch 1	Branch 2	Branch 3	Branch 4	Branch 5	Branch 1	Branch 2	Branch 3	Branch 4	Branch 5
Pb_208	3	4,1	1,7	7,2	/	2,2	3	1,3	5,1	/	27 %	27 %	24 %	29 %	/
Pb_218	2,5	8,3	4	/	/	1,9	7,1	/	/	/	24 %	14 %	/	/	/
Pb_156	7	5	4,3	/	/	5,2	3,5	3,2	/	/	26 %	30 %	26 %	/	/
Pb_303	8,9	3,3	3,7	/	/	6,9	2,8	2,9	/	/	22 %	15 %	22 %	/	/
Pb_279	6,8	4,3	4,5	3,9	/	5,5	3,4	3,3	3	/	19 %	21 %	27 %	23 %	/
Pb_132	4,6	3	8,3	/	/	3,6	2,2	6,3	/	/	22 %	27 %	24 %	/	/

Kera	Branch 1	Branch 2	Branch 3	Branch 4	Branch 5	Branch 1	Branch 2	Branch 3	Branch 4	Branch 5	Branch 1	Branch 2	Branch 3	Branch 4	Branch 5
Qp_39	3,4	2,4	5,8	/	/	2,6	1,9	4,6	/	/	24 %	21 %	21 %	/	/
Qp_18	7	4,4	2,3	/	/	5,5	3,4	1,6	/	/	21 %	23 %	30 %	/	/
Qp_20	2	3,7	6,5	/	/	1,5	2,9	5,2	/	/	25 %	22 %	20 %	/	/
Qp_19	3	4,8	1,4	/	/	2,5	3,7	1	/	/	17 %	23 %		/	/
Qp_35	3,9	4,2	5,5	/	/	3,1	3	4,5	/	/	21 %	29 %	18 %	/	/
Qp_23	2,4	2,6	5,2	/	/	2,1	2,3	4	/	/	13 %	12 %	23 %	/	/
Qp_27	3,4	2,5	3,8	/	/	2,3	1,8	3,1	/	/	32 %	28 %	18 %	/	/
Qp_37	6,8	4,7	4,8	/	/	5	3,5	3,7	/	/	26 %	26 %	23 %	/	/
Qp_41	6,5	4,7	4,6	/	/	5	3,2	3,3	/	/	23 %	32 %	28 %	/	/
Qp_14	1,6	2,8	5	/	/	1,1	1,9	4	/	/	31 %	32 %	20 %	/	/
Qp_22	6,2	5,2	3	/	/	4,7	4	2,1	/	/	24 %	23 %	30 %	/	/
Qp_24	7,1	3,8	4,3	/	/	5	3	3,3	/	/	30 %	21 %	23 %	/	/
Qp_53	3	5,5	5,2	/	/	2	4,1	4	/	/	33 %	25 %	23 %	/	/
Qp_16	1,2	5,5	5	/	/	1	4,2	4	/	/	17 %	24 %	20 %	/	/
Qp_9	2,9	2,6	/	/	/	2	1,9	/	/	/	31 %	27 %	/	/	/
  • Table 4 shows the number of samples taken from each tree (trunks and branches) and the number analysed.

**Table 4:** Number of Samples collected (cores and branches) and number of samples measured.

Site/plot	Name of tree	No core sampled	No branch sampled	No core analysed	No branches analysed
Kritsa_1	Cs120	2	6	2	6
Kritsa_1	Cs83	2	2	2	2
Kritsa_1	Cs51	2	5	2	2
Kritsa_1	Cs82	2	3	2	3
Kritsa_1	Cs42	2	3	2	3
Kritsa_2	Cs37	2	3	2	3
Kritsa_2	Cs13	2	3	2	3
Kritsa_2	Cs26	2	3	2	2
Kritsa_2	Cs35	2	3	2	2
Kritsa_2	Cs25	3	3	3	2
Kritsa_3	Cs52	2	3	2	2
Kritsa_3	Cs15	3	3	2	3
Kritsa_3	Cs21	2	3	2	3
Kritsa_3	Cs43	2	3	2	3
Kritsa_3	Cs26	2	3	2	2
Kroustas_0	Pb208	1	4	1	4
Kroustas_0	Pb156	1	3	1	3
Kroustas_0	Pb279	1	4	1	4
Kroustas_1	Pb330	1	3	1	3
Kroustas_1	Pb303	3	3	2	3
Kroustas_1	Pb162	2	4	2	4
Kroustas_1	Pb180	2	2	2	3
Kroustas_1	Pb170	2	3	2	2
Kroustas_2	Pb136	3	3	2	3
Kroustas_2	Pb46	1	3	0	3
Kroustas_2	Pb124	2	3	2	3
Kroustas_2	Pb218	2	3	2	2
Kroustas_2	Pb132	2	3	2	3
Kroustas_3	Pb133	2	3	2	2
Kroustas_3	Pb131	2	3	2	3
Kroustas_3	Pb94	2	3	2	3
Kroustas_3	Pb147	2	3	2	2
Kroustas_3	Pb148	2	3	2	3
Kera_1	Qp19dbh	2	3	2	3
Kera_1	Qp20dbh	2	3	2	3
Kera_1	Qp39dbh	2	3	2	3
Kera_1	Qp18dbh	2	3	1	3
Kera_1	Qp35dbh	2	3	1	3
Kera_2	Qp37dbh	2	3	2	3
Kera_2	Qp23dbh	2	3	2	3
Kera_2	Qp14dbh	2	3	2	3
Kera_2	Qp41dbh	2	3	2	2
Kera_2	Qp27dbh	2	3	2	3
Kera_3	Qp53dbh	/	3	0	3
Kera_3	Qp16dbh	2	3	2	3
Kera_3	Qp09dbh	2	3	2	2
Kera_3	Qp22dbh	2	3	2	3
Kera_3	Qp24dbh	2	3	2	0
  • Table 5. Table of individual cross-series for Turkish pine, with Student's *t*-test values, correlation coefficient, and overlap.

**Table 5:** Table of individual cross-series for Turkish pine with Student's *t*-test values, correlation coefficient and overlap.

	Rest	Pb132	Pb131	Pb124	Pb147	Pb208	Pb218	Pb136	Pb148	Pb94	Pb170	Pb330	Pb303
	CC TT OVL	CC TT OVL	CC TT OVL	CC TT OVL	CC TT OVL	CC TT OVL	CC TT OVL	CC TT OVL	CC TT OVL	CC TT OVL	CC TT OVL	CC TT OVL	CC TT OVL
Rest		0,69 6,9 54	0,72 9,8 92	0,74 9,3 71	0,70 7,1 56	0,67 8,6 93	0,58 5,6 64	0,57 5,4 62	0,57 7,6 120	0,59 6,9 93	0,56 7,4 120	0,55 5,9 85	0,52 5,5 84
Pb132	0,69 6,9 54		0,57 5,1 54	0,57 5,0 54	0,70 6,7 50	0,49 4,0 54	0,58 5,1 54	0,50 4,2 53	0,52 4,4 54	0,42 3,4 54	0,36 2,8 54	0,30 2,1 48	0,38 3,0 54
Pb131	0,72 9,8 92	0,57 5,1 54		0,58 6,0 71	0,58 5,2 56	0,58 6,8 91	0,63 6,4 64	0,45 3,9 62	0,58 6,8 92	0,48 5,0 86	0,44 4,6 92	0,41 4,0 80	0,46 4,7 84
Pb124	0,74 9,3 71	0,57 5,0 54	0,58 6,0 71		0,50 4,2 56	0,56 5,6 70	0,41 3,6 64	0,44 3,8 62	0,61 6,5 71	0,66 6,9 65	0,55 5,5 71	0,32 2,5 59	0,35 3,0 69
Pb147	0,70 7,1 56	0,70 6,7 50	0,58 5,2 56	0,50 4,2 56		0,57 5,1 55	0,50 4,2 56	0,50 4,0 49	0,49 4,2 56	0,40 3,1 51	0,33 2,6 56	0,49 3,6 44	0,35 2,7 54
Pb208	0,67 8,6 93	0,49 4,0 54	0,58 6,8 91	0,56 5,6 70	0,57 5,1 55		0,45 3,9 63	0,40 3,4 62	0,52 5,9 93	0,47 5,0 88	0,50 5,5 93	0,40 3,9 82	0,28 2,7 84
Pb218	0,58 5,6 64	0,58 5,1 54	0,63 6,4 64	0,41 3,6 64	0,50 4,2 56	0,45 3,9 63		0,33 2,6 57	0,37 3,1 64	*0,19 1,5 59	0,35 3,0 64	0,35 2,6 52	0,45 3,9 62
Pb136	0,57 5,4 62	0,50 4,2 53	0,45 3,9 62	0,44 3,8 62	0,50 4,0 49	0,40 3,4 62	0,33 2,6 57		0,34 2,8 62	0,42 3,5 61	0,34 2,8 62	0,41 3,3 57	0,42 3,6 62
Pb148	0,57 7,6 120	0,52 4,4 54	0,58 6,8 92	0,61 6,5 71	0,49 4,2 56	0,52 5,9 93	0,37 3,1 64	0,34 2,8 62		0,49 5,4 93	0,53 6,8 120	0,48 5,0 85	0,50 5,2 84
Pb94	0,59 6993	0,42 3,4 54	0,48 5,0 86	0,66 6,9 65	0,40 3,1 51	0,47 5,0 88	*0,19 1,5 59	0,42 3,5 61	0,49 5,4 93		0,39 4,1 93	0,33 3,2 84	0,24 2,2 80
Pb170	0,56 7,4 120	0,36 2,8 54	0,44 4,6 92	0,55 5,5 71	0,33 2,6 56	0,50 5,5 93	0,35 3,0 64	0,34 2,8 62	0,53 6,8 120	0,39 4,1 93		0,38 3,8 85	0,33 3,2 84
Pb330	0,55 5,9 85	0,30 2,1 48	0,41 4,0 80	0,32 2,5 59	0,49 3,6 44	0,40 3,9 82	0,35 2,6 52	0,41 3,3 57	0,48 5,0 85	0,33 3,2 84	0,38 3,8 85		0,52 5,2 74
Pb303	0,52 5,5 84	0,38 3,0 54	0,46 4,7 84	0,35 3,0 69	0,35 2,7 54	0,28 2,7 84	0,45 3,9 62	0,42 3,6 62	0,50 5,2 84	0,24 2,2 80	0,33 3,2 84	0,52 5,2 74
  • Table 6. Table of individual cross-series for Downy oak, with Student's *t*-test values, correlation coefficient, and overlap.

**Table 6:** Table of individual cross-series for Downy oak with Student's *t*-test values, correlation coefficient and overlap.

	Rest	Qb14	Qb16	Qb22	Qb24	Qb27	Qb37	Qb41
	CC TT OVL	CC TT OVL	CC TT OVL	CC TT OVL	CC TT OVL	CC TT OVL	CC TT OVL	CC TT OVL
Rest		0,67 3,9 21	0,66 5,1 36	0,76 6,6 34	0,56 4,3 42	0,82 7,3 27	0,46 3,3 42	0,55 3,9 37
Qb14	0,67 3,9 21			0,53 2,7 20	0,65 3,6 20	0,62 3,4 20	0,42 1,9 20	0,44 2,2 21
Qb16	0,66 5,1 36			0,68 4,7 28	0,53 3,7 36	0,57 3,1 21	0,28 1,7 36	0,23 1,2 30
Qb22	0,76 6,6 34	0,53 2,7 20	0,68 4,7 28		0,50 3,2 34	0,69 4,7 27	0,42 2,6 34	0,50 3,3 34
Qb24	0,56 4,3 42	0,65 3,6 20	0,53 3,7 36	0,50 3,2 34		0,61 3,9 27	0,23 1,5 42	0,37 2,3 36
Qb27	0,82 7,3 27	0,62 3,4 20	0,57 3,1 21	0,69 4,7 27	0,61 3,9 27		0,49 2,8 27	0,61 3,9 27
Qb37	0,46 3,3 42	0,42 1,9 20	0,28 1,7 36	0,42 2,6 34	0,23 1,5 42	0,49 2,8 27		0,50 3,3 36
Qb41	0,55 3,9 37	0,44 2,2 21	0,23 1,2 30	0,50 3,3 34	0,37 2,3 36	0,61 3,9 27	0,50 3,3 36
  • Table 7 shows the lower and upper hinges, as well as the lower and upper whiskers, of ring-width distributions for the three reference groups by branch and trunk. It also includes the results of the Wilcoxon test for each species.

**Table 7:** The lower and upper hinges, as well as the lower and upper whiskers, of ring width distributions for the three reference groups by branch and trunk. It also includes the results of the Wilcoxon test for each species.
  • Table 8 consists of three references to growth patterns, including the country, the full name of the locality and forest, the forest type, the station code, the altitude, the taxon, the tree code, the data type (trunk or branch), the coring height for the cores, the organ age, the years of ring formation, the ring widths, the cumulative radius of the ring widths, the diameter class associated with the organ, the authors of the measurements and sampling, and the wood type (normal or reaction wood).
• **(3)** Figures Folder(.pdf): This folder contains the 4 figures shown in this data paper.
  • Fig. 1: Location of forests, plots, and sampled trees.

**Fig. 1:**
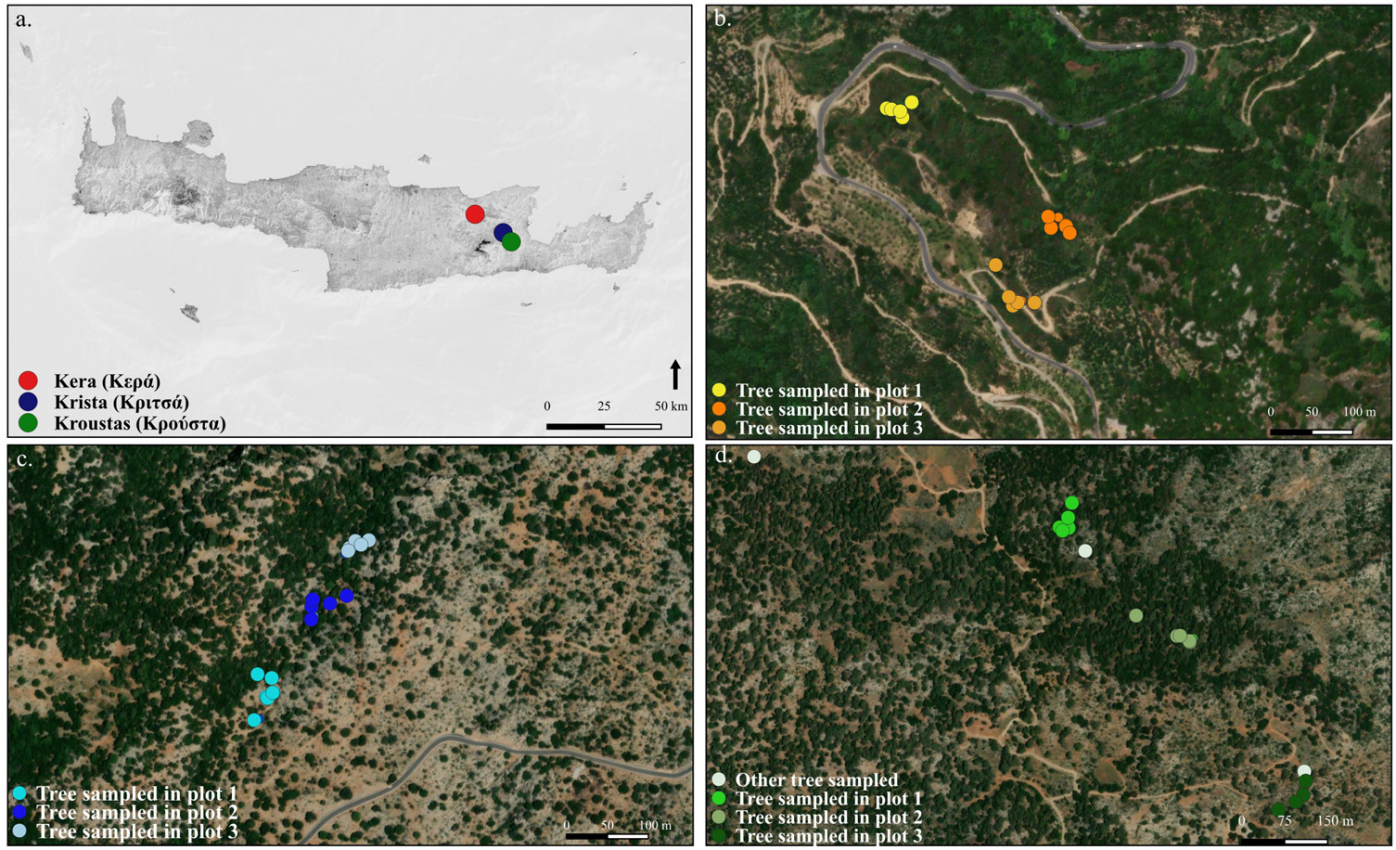
Localisation of forests, plots and tree sampling: a. Map of Crete showing the location of the three forest sites; b. Kera (Downy oak) with its three sampled plots; c. Kritsa (Mediterranean cypress) with its three sampled plots; d. Kroustas (Turkish pine) with its three sampled plots. For b., c., d., each colour corresponds to a sampled plot, and each point represents an individual sampled tree.
  • Fig. 2: Crossdating and the mean of single chronologies for Turkish pine trunk (with only samples with CC>0,4).

**Fig. 2:**
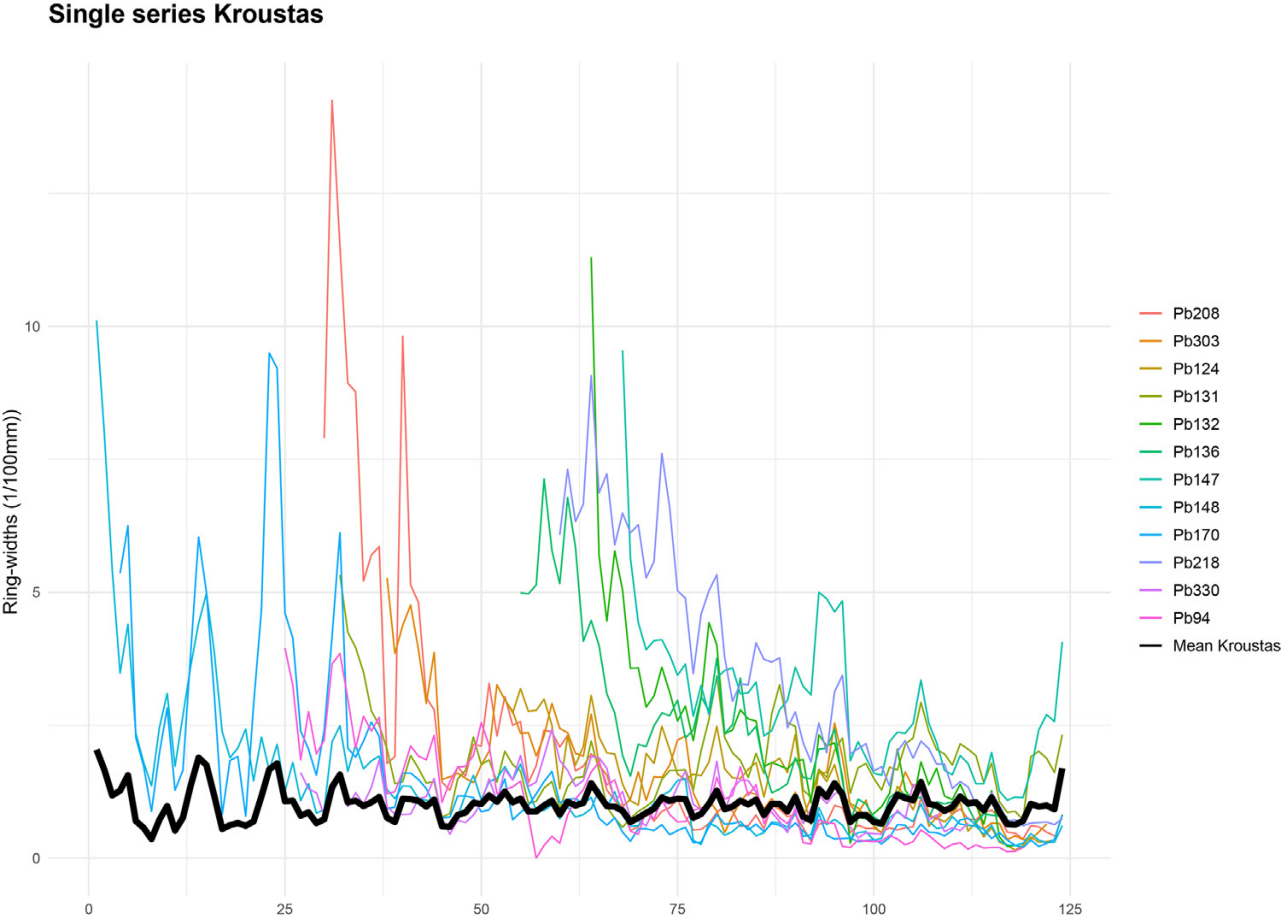
Crossdating and mean chronology for Turkish pine trunks (only samples with a correlation coefficient [CC] > 0.4 are included). The mean chronology is shown in black, and individual series in other colors.
  • Fig. 3: Crossdating and the mean of single chronologies for Downy oak trunk (with only samples with CC>0,4).

**Fig. 3:**
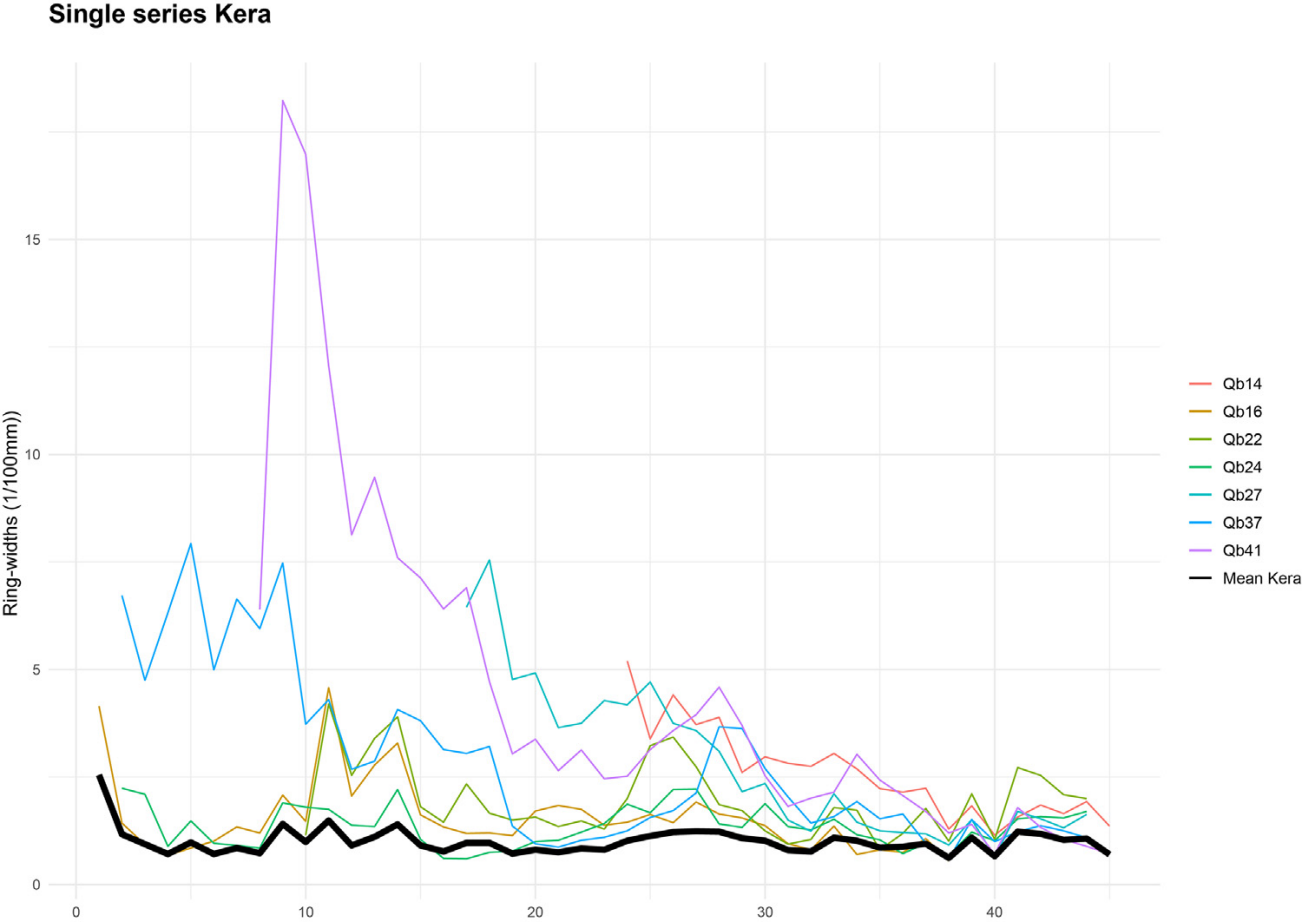
Crossdating and mean chronology for Downy oak trunks (only samples with a correlation coefficient [CC] > 0.4 are included). The mean chronology is shown in black, and individual series in other colors.
  • Fig. 4: Box plots show the distribution of ring widths (trunk and branch) for each species.

**Fig. 4:**
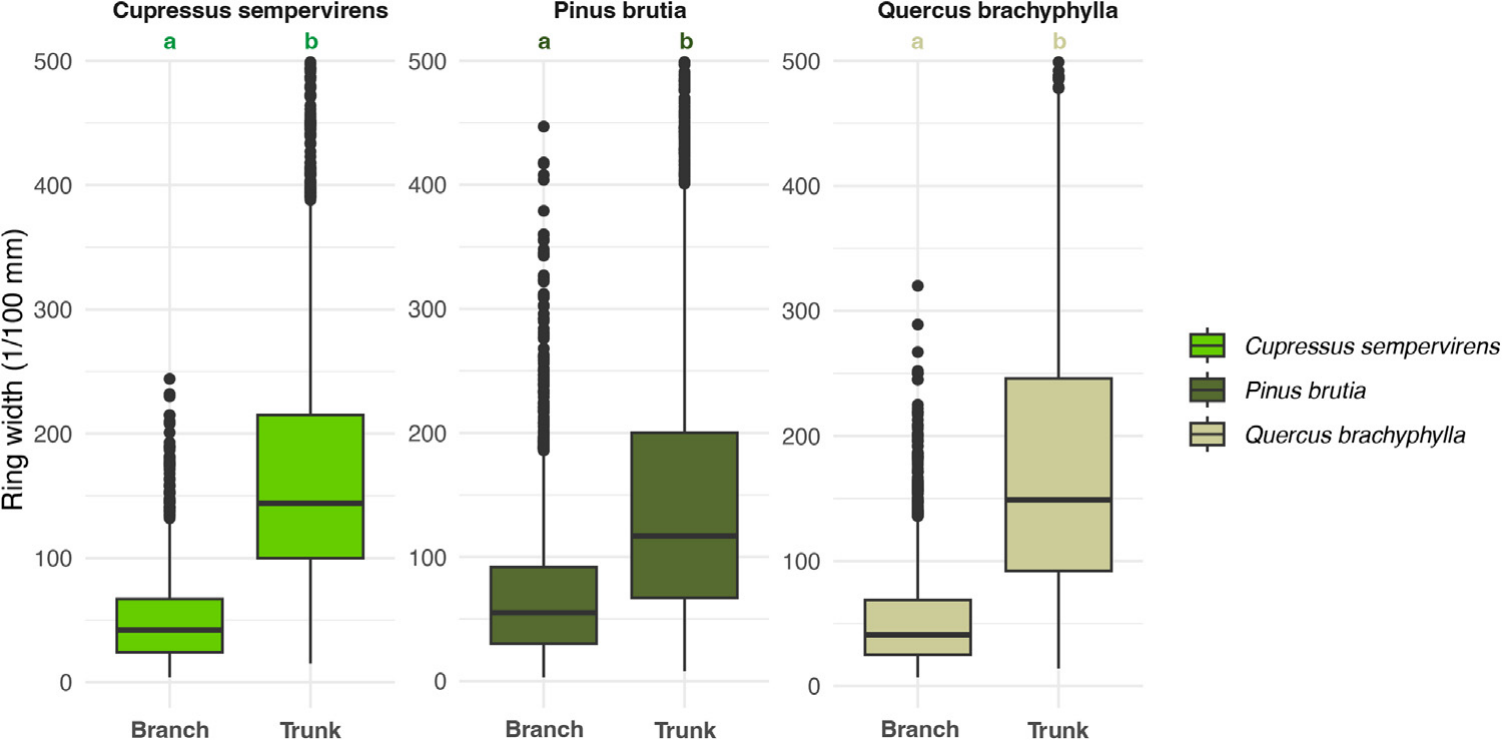
Box plots showing the distribution of ring widths for each species and for branch and trunk wood; we kept only ring width <500 (1/100 mm) for plot visibility because of the presence of many outliers.
• **(4) R Script Folder:** This folder contains R scripts for reading and performing various operations on the ring-width measurements of the references and archaeological materials, using the DplR; ggplot2; dplyr; readxl; tidyr; openxlsx; tidyverse; caret; pROC packages. R version: 4.4.2 (2024–10–31 ucrt)
  • **Statistics.R:** provides an R script to graphically represent the differences in growth patterns between the trunk and branches for the three species, and to statistically assess whether the growth patterns differ significantly.
  • **PredictWoodOrigin.R:** provides a practical tool for users wishing to apply the reference model to their own tree-ring width data. It includes all necessary steps to preprocess input data, apply the trained logistic regression model, and classify each observation as originating from either branch-wood or trunk-wood. Users can input their own measurements−such as archaeological wood or charcoal ring-width data− for one of the three species included in the reference collection. The script outputs predicted anatomical origins along with associated probabilities, enabling researchers to evaluate the classification confidence. This tool is intended to support archaeological analyses aiming to reconstruct past wood-use practices, such as fuel selection or woodland exploitation, based on the anatomical origin of wood remains.
• **(5) Supplementary file:** A .txt file providing information on the environmental history and distribution areas of the species studied.
• **(6) Read Me File:** A .txt file providing explanations for easily navigating and using all data provided in this directory.

## 4 Experimental Design, Materials and Methods

### 4.1 Materials & methods

The reference sites were established in three Cretan forests following the protocols developed by Dufraisse et al. [15], and subsequently applied by Picornell et al. [16] and Alcolea et al. [17]. Forests were selected based on their species composition and their proximity to the archaeological sites under investigation by the TiMMA project, all located in the east-central part of Crete (Zakros, Malia, and Phaistos). The sampling campaign was carried out in April 2023 and April 2024.

For each reference species, a dedicated forest stand was identified (Fig. 1; Table 1):

• Kroustas Forest for Turkish pine (35.121910, 25.633253);
• Kritsa Forest for Mediterranean cypress (35.157856, 25.594416);
• Kera Forest for Downy oak (35.230220, 25.458989).

The first campaign consisted of an exploratory phase aimed at identifying suitable forest stands and assessing the feasibility of the sampling protocol on a very limited number of individuals. This preliminary work laid the groundwork for the full-scale sampling carried out during the second campaign, which took place after contacting the relevant authorities.

### 4.2 Sampling strategies

Sampling in eastern Crete could not follow a straightforward altitudinal gradient due to the severe contraction of the forest areas currently occupied by the three target species, mainly as a result of long-term human pressure, particularly for Mediterranean cypress and Downy oak, whose current distribution is highly fragmented in this region. A complementary study following an altitudinal and ecological gradient in western Crete, where both taxa occur more continuously, could help refine and extend the present reference dataset.

Our sampling design was adapted from the dendro- and anthraco-typological framework developed in recent work (e.g [16]), with a field protocol adjusted to optimise representativeness relative to effort. Three plots per species and five trees per plot constituted a balanced compromise between capturing within-stand variability and ensuring feasible laboratory processing.

For each of the three target species, three plots of approximately 1 hectare were established within representative stands: at 780–890 m a.s.l. for Turkish pine in the Kroustas Forest, 870–950 m a.s.l. for Mediterranean cypress in the Kritsa Forest, and 540–560 m a.s.l. for downy oak in the Kera woodland (Table 1, Table 2). The Kroustas and Kritsa forests preserve numerous archaeological remains from the Minoan period, together with a continuous and diverse record of agrosilvopastoral land use across Late Holocene cultural phases [18,19,20]. At plot level, we also documented evidence of human management and past tree uses (e.g. coppice systems, pollarding, wood-pasture, *tèdes*).

In each plot, five individual trees were selected for sampling, for a total of 48 trees across all sites (3 additional trees were tested for Turkish pines—corresponding to white dotes on the map (Fig. 1)). For each tree, two increment cores were extracted from the trunk at breast height (about 1.30 m) using a Pressler borer. In addition, three branch slices of varying diameters were collected from each tree using a pruning saw with a telescopic handle; branches were cut as close as possible to the branch collar. We preferably selected recently dead branches that were still present in the tree crown. To account for age-dependent variability in growth patterns, we have selected a range of tree diameters from 9 to over 20 cm. In total, the sampling yielded 93 trunk cores and 150 branch slices. In addition, we recorded: (i) the diameter at breast height, measured at about 1.3 m above ground level; (ii) the total tree height, defined as the distance from the ground to the terminal bud; and (iii) the competition index, calculated as the number of neighbouring trees whose upper branches overlap or intermingle with those of the target tree (Table 2).

### 4.3 Samples preparation and dendrochronological analysis

All samples were first air-dried in the laboratory. Trunk cores were mounted on wooden holders to facilitate handling and measurement. Both trunk cores and branch slices were then progressively sanded using a belt sander with abrasive papers ranging from 100 to 500 grit in order to enhance the visibility of growth-ring boundaries. Ring widths were measured to a precision of 0.01 mm using a Lintab-6 measuring table connected to TSAP-Win software [21]. The diameters (on normal wood) of the branch slices were carefully recorded to document the variability in branch size (Table 3).

To ensure accurate ring counts and account for possible measurement biases, particular attention was given to the identification of the first and last growth rings in each sample.

For each core sample, the first measured ring generally corresponds to the last complete and clearly identifiable annual ring formed by the tree, comprising both earlywood and latewood. However, this outermost ring does not always represent the calendar year of sampling, as the final growth ring, or, in some cases, the last few rings, may have been lost during coring due to mechanical damage, such as crushing or detachment during extraction. Similarly, the innermost rings, potentially including the pith, may be missing as a result of heartwood decay or off-centre sampling. Consequently, the counted number of rings does not necessarily provide a reliable estimate of the tree’s total age.

A total of 87 of 93 trunk cores were measured (31/32 for Mediterranean cypress; 30/33 for Turkish pine; 26/28 for Downy oak), and 134 out of 150 branch samples were measured (41/49 for Mediterranean cypress; 53/56 for Turkish pine; 40/45 for Downy oak) (Table 4).

The measured individual series were then statistically processed and crossdated to build the reference chronologies. In the C-DENDRO software, the series were automatically normalised using the P2yrsL method (Proportion of last two years growth limited). The accuracy of the crossdating was assessed using Student's *t*-test (t) (threshold ≥ 4), the correlation coefficient r with a threshold of 0.4, and the χ² test, indicating statistically significant agreement based on overlap and degrees of freedom [22,23] (Table 5, Table 6). For the Turkish pine and Downy oak forests, an average chronology was produced (Figs. 2,3). The resulting mean chronology comprises 12 pine tree trunks out of 18 and 7 oak trunks out of 14. For Mediterranean cypress, the individual chronologies could not be crossdated (see Limitations section).

### 4.4 Experimental carbonisation protocol and post-treatment

After ring-width measurements, branch samples were air-dried again for an additional time. They were then carbonised in a Nabertherm calcination furnace at a target temperature of 500 °C. Due to the limited internal volume of the furnace and the risk of temperature fluctuations exceeding the set point, the number of samples per charring was carefully controlled. Depending on their size, between one and three individual tree samples (up to a maximum of nine branch slices) were carbonised per session, with one carbonisation carried out per day.

A placement diagram was created for each carbonisation session to prevent any confusion during post-charring retrieval.

To prevent combustion and ensure proper carbonisation, the oxygen supply was limited by two means: (1) the use of a closed laboratory carbonisation furnace, and (2) the systematic wrapping of each branch sample in aluminium foil. The wrapped samples were placed in a covered porcelain container and further insulated with a layer of sand. This setup creates a locally oxygen-poor environment, preventing ignition during the thermal ramp-up. As the ignition threshold of wood in the presence of oxygen is typically reached between 280 °C and 300 °C, such precautions are essential to avoid partial or total combustion and to obtain homogenous charcoal suitable for analysis.

Once the samples were arranged, the following programme was initiated:

• Starting temperature: ambient
• S01: ramp to 300 °C in 1 hour
• S02: ramp to 500 °C in 1 hour
• S03: hold at 500 °C for 30 min.

This protocol was selected to produce well-formed charcoal with a high carbon content, as typically required for reference datasets. Although partial carbonisation can occur from ∼350 °C, temperatures between 400 and 500 °C are generally necessary to ensure a stable and homogeneous carbon structure. The upper limit of 500 °C was chosen to avoid excessive shrinkage, which is commonly observed above 700 °C and may compromise the morphological integrity of the samples. This protocol follows routine procedures used in anthracological reference material production.

At the end of the programme, the furnace was left to cool passively to approximately 200 °C before it could be safely opened. Once cooled, the charcoal samples were carefully retrieved. The charcoal samples were individually stored in grip-seal bags and labelled.

The diameter of each charred branch disc was then measured again, and the percentage of radial shrinkage was calculated by comparing the diameters before and after charring. Average shrinkage values were then computed for each species (Table 3).

### 4.5 Testing for differences in tree-ring width between trunk and branch wood (R.script 1)

A key challenge in contemporary archaeological studies is to improve the characterisation of past practices, particularly by identifying the differential use of various parts of the tree, including trunk and branch wood. In this context, we assessed the feasibility of distinguishing the anatomical origin of wood (trunk vs. branch) based on tree-ring width.

We first tested the distribution of ring widths using the Shapiro-Wilk test. For the full dataset, the distributions deviated from normality (Table 7). Accordingly, we applied a Wilcoxon test to compare ring widths between trunk and branch wood samples for each of the three different species. The results indicate statistically significant differences in ring width between trunk and branch wood across all species (*p* < 2.2e-16) (Table 7, Fig. 4).

These results suggest that tree-ring width is a robust indicator of the anatomical origin of the wood. This approach offers promising opportunities for enhancing the interpretation of archaeological charcoal assemblages and for reconstructing the technical decisions involved in past tree-harvesting practices.

### 4.6 Testing value of the data in archaeological context (R.script 2)

To illustrate a potential use of the reference datasets, we provide a R script that builds and evaluates a logistic regression model based on a random subset of the tree-ring-width data. This model aims to predict the origin of a wood sample−whether it derives from a branch or a trunk−using individual ring-width values as predictors. The model is trained and tested on a stratified sample of the dataset, covering the three species included in the reference collection. The predictive performance of the model is assessed using sensitivity (true positive rate), specificity (true negative rate), and the area under the receiver operating characteristic curve (AUC-ROC). The ROC curve is also provided to visualise the trade-off between sensitivity and specificity across decision thresholds. Results show that ring width alone can effectively discriminate between branch-wood and trunk-wood in two of the three species: oak and cypress. For pine, the model could be refined and improved by adding new reference data. This approach is particularly relevant for archaeological applications, where determining the origin of charcoal or wood remains is often critical for reconstructing past woodland management, fuel selection, or wood-use strategies. This script can be reused on any sample of archaeological origin, as long as the user formats their dataset correctly, in accordance with the data associated with this datapaper.

### 4.7 Prerequisites for applying the tool to archaeological charcoal

Before applying the protocol to distinguish between branch wood and trunk wood in archaeological charcoal fragments, it is essential to reconstruct the original diameter of the wood from which the charcoal was derived. This estimated pre-carbonisation diameter is crucial for accurately interpreting ring-width patterns and determining the anatomical origin of the sample. The reconstruction must follow established procedures as described by Dufraisse and Garcia [24], Paradis-Grenouillet et al. [25], and Dufraisse et al. [15,26], ideally using the open-access tools available on the ANR DENDRAC platform (https://dendrac.mnhn.fr).

For a fragment to be considered suitable for analysis, it must preserve at least three consecutive growth rings and exceed 3 mm in both radial and transverse directions.

Once the initial diameter has been reconstructed, a correction must be applied to account for shrinkage. Mean shrinkage rates vary by species: 22 % for Mediterranean cypress, 23 % for Turkish pine, and 24 % for Downy oak, based on the values presented in Table 3. In addition, if the reconstructed diameter exceeds 10 cm, a further correction of 17 % should be applied, following the recommendation of Dufraisse and Garcia [24] and Dufraisse et al. [15]. Lastly, ring-width measurements from archaeological charcoal samples should be recorded in an Excel spreadsheet, formatted in accordance with Table 8 (available here: https://data.indores.fr:443/api/access/datafile/29440) to allow comparison with the reference datasets. We also kindly remind users of the two R scripts to preserve the structure of the original table, ensuring that all required columns are present and that column names remain unchanged.

## Limitations

The high tannin content in Downy oak and the resin content in Turkish pine caused some samples (*n* = 4) to adhere to the increment borer, resulting in the clogging of two augers. In addition, several cores twisted during the drying process, rendering them unusable (Table 4).

Branch chronologies and cypress chronologies in this dataset did not yield reliable crossdating results and therefore cannot be directly used as dendrochronological references. Crossdating of branch samples was not feasible due to the lack of sufficiently long and consistent ring series. Cypress trees, located at low to medium altitude (<1000 m a.s.l.) are known to exhibit frequent growth anomalies such as false rings, which hinder reliable crossdating [[10], [27], [28], [29]]. Most false rings were identified by comparing the two trunk cores or by analysing distinct radii in branch slices. However, undetected anomalies may still be present, and crossdating was therefore not pursued so as to avoid compromising the rest of the study. Users who wish to use these chronologies for dating purposes should first realign them against independently validated reference chronologies. In contrast, the oak and pine chronologies provided in this dataset show consistent internal coherence and can be reused as dendrochronological references without additional adjustment.

Another limitation of this dataset is that, due to the restricted current distribution of Downy oak, sampling was constrained to a limited geographical and environmental range, and sampling for Mediterranean cypress was also restricted to a specific geographic area. As a result, the full variability of ring-width patterns in branch-wood and trunk-wood across broader environmental gradients may not be fully captured. Users should therefore consider this constraint when applying the reference data to samples originating from markedly different ecological contexts, particularly those outside the current range of the species. In contrast, the Turkish pine reference dataset incorporates greater variability. Trees in the first two plots showed signs of past disturbance, such as stem scars, and were located either on the edge of old terraces or in more open, scattered formations. The third plot, by contrast, was situated within a denser pine forest.

The final limitation concerns the reuse of reference datasets. It is crucial to assess their replicability and geographic relevance to ensure their applicability beyond their original context.

While successful replication would allow for broader regional interpretations, it requires careful validation. Tree-ring growth patterns and shrinkage rates can vary according to local climatic and geological conditions, so a reference dataset should ideally reflect the environment of the specific study site as closely as possible.

To test replicability, samples—both cores and branches—should be collected from the same taxa in various locations, including other Aegean islands and mainland Greece. These samples can then be analysed and compared with the Cretan dataset to evaluate the consistency of growth patterns across regions.

## Ethics Statement

The authors certify that they have read and complied with the ethical requirements for publication in Data in Brief and confirm that the current work does not involve human subjects, animal experiments or data collected on social media platforms.

## CRediT authorship contribution statement

**Léane Levillain:** Conceptualization, Methodology, Validation, Formal analysis, Investigation, Data curation, Writing – original draft, Visualization. **Mélanie Saulnier:** Methodology, Validation, Formal analysis, Writing – original draft, Visualization. **Alexa Dufraisse:** Methodology, Writing – review & editing. **Laurent Larrieu:** Methodology, Validation, Investigation, Writing – review & editing. **Maria Ntinou:** Validation, Writing – review & editing, Project administration. **Vanessa Py-Saragaglia:** Conceptualization, Methodology, Validation, Investigation, Writing – original draft, Supervision, Project administration.

## Data Availability

DataverseWood growth pattern reference datasets for Pinus brutia Ten., Cupressus sempervirens L., and Quercus pubescens Willd. subsp. pubescens from Crete for dendro- and anthraco-typological analysis (Reference data).

## Appendix Supplementary materials

Image, application 1
