# Supplementary material for: Trunk- and branch- data derived radial increments of Pinus brutia Ten., Cupressus sempervirens L., and Quercus pubescens Willd. subsp. pubescens from Crete for dendro- and anthraco-typological analysis
Source: Data Brief. 2025 Nov 23;64:112307. doi: 10.1016/j.dib.2025.112307 (PMC12721281; doi:10.1016/j.dib.2025.112307)
Supplement: Supplementary file 1 [file mmc1.docx]

SUPPLEMENTARY FILE N°1a:

The TiMMA project – *Timber in Minoan and Mycenaean Architecture* (ANR21-CE27-0029; <https://timma.efa.gr/s/timma/page/the-timma-project>) – is coordinated by Sylvie Rougier-Blanc (UPEC-CRHEC). Work Package 1, DATA-WOOD, led by Maria Ntinou, Clémence Pagnoux and Vanessa Py-Saragaglia, develops and applies analytical methods such as dendro-typology (Billamboz, 2011, 2014) and anthraco-typology (Dufraisse et al., 2018; Picornell et al., 2020; Alcolea et al., 2021). The latter compares growth-ring patterns in archaeological charcoals with those in experimentally charred modern references. Both approaches rely on robust datasets that capture ring-width variability and quantify shrinkage effects induced by carbonisation.

SUPPLEMENTARY FILE N°1b:

Turkish pine (*Pinus brutia* Ten. 1811), Mediterranean cypress (*Cupressus sempervirens* L. 1753), and Downy oak (*Quercus pubescens* Willd subsp. *Pubescens,* 1796): (i) are repeatedly attested in anthracological assemblages from Aegean Bronze Age contexts on Crete (Shaw, 2009; Moody, 2012; Ntinou et al., 2019; Ntinou, 2022); in the case of cypress, the species is also explicitly mentioned in Linear B tablets of the Mycenaean period as wood for chariot wheels (Rougier-Blanc, 2020, p. 190-193, cf. PY Sa 488, Rougier-Blanc, 2021, pp. 55-62); (ii) are still present today in fairly dense stands suitable for dendro-sampling; (iii) are anatomically well suited to ring-width analysis, with clearly visible growth rings; and (iv) are representative of both softwoods and hardwoods widely used in construction.

Palynological evidence from several locations on Crete provides a picture of the vegetation cover on the island during the Holocene. The pollen records indicate the importance of mixed deciduous and evergreen oak woodlands and maquis on western Crete until the 5^th^ millennium BCE. On eastern Crete during the same period, phrygana and maquis vegetation with *Olea* dominated the lowlands, while scarce, patchy pine and oak forests existed in protected locations. During the Bronze Age (3100-1100 BCE) Mediterranean maquis and shrub communities increased, and *Olea* dominated the landscape (Bottema, 1980; Bottema and Sarpaki, 2003; Cañellas-Boltà et al., 2018; Ghilardi et al., 2018; Jouffroy-Bapicot et al., 2021). The spread of olive cultivation likely contributed to the decline of mixed woodlands during the Bronze Age, particularly in the thermo- and meso-Mediterranean belts.  The use of pine, deciduous oak and cypress wood is documented in anthracological assemblages from several Bronze Age sites on Crete (Rackham 1972; Follieri-Coccolini, 1986; Shay and Shay, 1995; Fiorentino and Solinas, 2006; Ntinou et al., 2019, 2025).

Today, Turkish pine predominantly forms forests in mountainous regions of both western and eastern Crete, mainly in the White, Lassithi and Siteia Mountains, but it can also grow at lower altitudes (Rackham and Moody, 1996; EUFORGEN). Downy oak is now primarily distributed in the western part of the island, while in the eastern part it is highly localised, mostly occurring above 500 m asl. There, it typically grows on former cultivated terraces and in hedgerows, where it was often managed through pollarding (Rackham and Moody, 1996, p. 65). Except in the White Mountains, the current distribution of Mediterranean cypress is similarly restricted, with its range now limited to a few fragmented areas, most notably in the Lassithi Mountains, which represent one of its main refugia in eastern Crete (Rackham and Moody, 1996, p.60; EUFORGEN). The Holocene history of cypress in Crete has long been difficult to assess due to the taxon’s low pollen production and morphological ambiguity with other Cupressaceae in palynological records (Bottema and Sarpaki, 2003). However, anthracological data from Neolithic Knossos, Minoan Phaistos, the Kamilari tholos tomb, and Late-Minoan II-III Kommos provide direct evidence for the early and long-standing use of cypress wood (Follieri and Coccolini, 1979; Shay and Shay, 1995; Badal and Ntinou, 2013;  Ntinou et al., 2019). Based on these findings, the authors suggest that cypress may have been locally available, forming stands in uncultivated lowland areas at the foot of the slopes, as well as at higher elevations in the inland mountains. While its ecological role and distribution remain unclear for this early period, its increasing presence in later historical landscapes likely may reflect both natural persistence and human-driven selection, particularly within open woodlands and culturally managed environments (Rackham and Moody, 1996). More recently, the regeneration of this species has been observed to be favoured by the abandonment of traditional agro-sylvo-pastoral practices (Rackham, 1990).

- - 1. Alcolea, M., Dufraisse, A., Royo, M., Mazo, C., de Luis, M., Longares, L.A., Utrilla, P., Fábregas, R., 2021. Dendro-anthracological tools applied to Scots type pine forests exploitation as fuel during the Mesolithic-Neolithic transition in the southern central pre-Pyrenees (Spain). *Quaternary International* 593–594, pp. 332–345.<https://doi.org/10.1016/j.quaint.2020.10.029>
    2. Badal, E., Ntinou, M., 2013. Wood Charcoal Analysis: The Local Vegetation, in: Efstratiou, N., Karetsou, A., Ntinou, M. (Eds.), *Neolithic Settlement of Knossos in Crete: New Evidence for the Early Occupation of Crete and the Aegean Islands*, Prehistory Monographs. INSTAP Academic Press, pp. 95–118.<https://doi.org/10.2307/j.ctt5vj96p>
    3. Billamboz, A., 2011. Applying dendro-typology to large timber series, in: Fraiture, P. (Ed.), *Tree rings, art, archaeology: proceedings of an international conference*, Royal Institute for Cultural Heritage, Brussels, pp. 177–188.
    4. Billamboz, A., 2014. Regional patterns of settlement and woodland developments: Dendroarchaeology in the Neolithic pile-dwellings on Lake Constance (Germany). *The Holocene* 24(10), pp. 1278–1287.<https://doi.org/10.1177/0959683614540956>
    5. Bottema, S., 1980. Palynological investigation on Crete. *Review of Palaeobotany and Palynology* 31, pp. 193–217.<https://doi.org/10.1016/0034-6667(80)90027-5>
    6. Bottema, S., Sarpaki, A., 2003. Environmental change in Crete: a 9000-year record of Holocene vegetation history and the effect of the Santorini eruption. *The Holocene* 13, pp. 733–749.<https://doi.org/10.1191/0959683603hl659rp>
    7. Cañellas-Boltà, N., Riera-Mora, S., Orengo, H.A., Livarda, A., Knappett, C., 2018. Human management and landscape changes at Palaikastro (Eastern Crete) from the Late Neolithic to the Early Minoan period. *Quaternary Science Reviews* 183, pp. 59–75.<https://doi.org/10.1016/j.quascirev.2018.01.010>
    8. Dufraisse, A., Coubray, S., Girardclos, O., Nocus, N., Lemoine, M., Dupouey, J.-L., Marguerie, D., 2018. Anthraco-Typology as a Key Approach to Past Firewood Exploitation and Woodland Management Reconstructions. Dendrological Reference Dataset Modelling with Dendro-Anthracological Tools. *Quaternary International* 463, pp. 232–249.<https://doi.org/10.1016/j.quaint.2017.03.065>
    9. Fiorentino, G., Solinas, F., 2006. Carboni e carporesti dal palazzo di Monastiraki, in: Kanta, A., Marazzi, M. (Eds.), *Monastiraki I Campagne 2002–2004*. Fondazione Scavolini, Naples, pp. 123–129 (Quaderni della Fondazione Scavolini, Serie Beni Culturali 4).
    10. Follieri, M., Coccolini, G.B.L., 1979. Travi carbonizzate del palazzo Minoico di Festos (Creta). *Annuario della Scuola Archeologica di Atene e delle Missioni Italiane in Oriente* 41–42, pp. 173–185.
    11. Ghilardi, M., Psomiadis, D., Andrieu-Ponel, V., Colleu, M., Sotiropoulos, P., Longo, F., Rossi, A., Amato, V., Gasse, F., Sinibaldi, L., Renard, M., Bicket, A., Delanghe, D., Demory, F., Fleury, J., 2018. First evidence of a lake at Ancient Phaistos (Messara Plain, South-Central Crete, Greece): Reconstructing paleoenvironments and differentiating the roles of human land-use and paleoclimate from Minoan to Roman times. *The Holocene* 28, pp. 1225–1244.<https://doi.org/10.1177/0959683618771473>
    12. Jouffroy-Bapicot, I., Pedrotta, T., Debret, M., Field, S., Sulpizio, R., Zanchetta, G., Sabatier, P., Roberts, N., Tinner, W., Walsh, K., Vannière, B., 2021. Olive groves around the lake. A ten-thousand-year history of a Cretan landscape (Greece) reveals the dominant role of humans in making this Mediterranean ecosystem. Quaternary Science Reviews 267, 107072.<https://doi.org/10.1016/j.quascirev.2021.107072>
    13. Moody, J., 2012. Hinterlands and hinterseas: resources and production zones in Bronze Age and Iron Age Crete. *British School at Athens Studies* 20, pp. 233–271.
    14. Ntinou, M., Picornell-Gelabert, Ll., Apostolakou, V., Brogan, T., Livarda, A., Sofianou, C.H., Soles, J., 2025. Woodlands, tree management, and fuel economy in Bronze Age eastern Crete: An anthracological approach. *Journal of Archaeological Science: Reports* 62, 105010.<https://doi.org/10.1016/j.jasrep.2025.105010>
    15. Ntinou, M., Rackham, O., Moody, J.A., Ważny, T., 2019. The wood charcoal remains from Kamilari Tholos A, in: Girella, L., Caloi, I. (Eds.), *Kamilari, Una necropoli di tombe a tholos nella Messarà (Creta)*. *Monografie della Scuola Archeologica di Atene e delle Missioni Italiane in Oriente* XXIX, pp. 639–646.
    16. Ntinou, M., 2022. Wood Charcoal, in: *Mochlos IVA: Period III. The House of the Metal Merchant and Other Buildings in the Neopalatial Town*, *Prehistory Monographs*. INSTAP Academic Press, Philadelphia, pp. 487–493.
    17. Picornell-Gelabert, L., Servera-Vives, G., Carrión Marco, Y., Burjachs, F., Currás, A., Llergo, Y., Dufraisse, A., De Luís Arrillaga, M., Mus Amézquita, M., 2020. Late Holocene Aleppo pine (*Pinus halepensis* Miller) woodlands in Mallorca (Balearic Islands, Western Mediterranean): Investigation of their distribution and the role of human management based on anthracological, dendro-anthracological and archaeopalynological data. *Quaternary International*, *Anthracology: Charcoal Science in Archaeology and Palaeoecology* 593–594, pp. 346–363.<https://doi.org/10.1016/j.quaint.2020.11.006>
    18. Rackham, O., Moody, J.A., 1996. *The making of the Cretan landscape*. Manchester University Press, Manchester ; New York.
    19. Rackham, O., 1990. *The last forest*. London, Dent.
    20. Rackham, O., 1972. Appendix III. Charcoal and plaster impressions, in: *Myrtos: An Early Bronze Age Settlement in Crete*. The Alden Press, Oxford, pp. 299–304.
    21. Rougier-Blanc, S., 2020. Premières remarques sur le matériau bois dans les tablettes en linéaire B et chez Homère, in: Guieu-Coppolani, A., Werlings, M.-J. and Zurbach, J., *Le pouvoir et la parole*. Mélanges en mémoire de Pierre Carlier, ADRA, Nancy-Paris, p. 173-201.
    22. Rougier-Blanc, S., 2021. Le bois dans l’architecture à l'époque mycénienne et au Premier Âge du Fer en Grèce continentale : technique et société. Volume V. Mémoire Inédit. Première partie, in: Rougier-Blanc, *Habitat, littérature et société en Grèce de l'Âge du Bronze Récent aux Sévères : Réalités et représentations.* [HDR], University of Lyon.
    23. Shaw, J., 2009. *Minoan architecture: Materials and techniques*, *Studi di Archeologia Cretese*. Bottega d’Erasmo, Padoue.
    24. Shay, C.T., Shay, J.M., with Frego, K.A. and Zwiazek, J., 1995. The modern flora and plant remains from Bronze Age deposits at Kommos, in: Shaw, J.W., Shaw, M.C. (Eds.), *Kommos I: The Kommos Region and Houses of the Minoan Town, Part 1. The Kommos Region, Ecology, and the Minoan Industries*. Princeton, pp. 91–162.
